# Upper Body Physical Rehabilitation for Children with Ataxia through IMU-Based Exergame

**DOI:** 10.3390/jcm11041065

**Published:** 2022-02-18

**Authors:** Alberto Romano, Martina Favetta, Susanna Summa, Tommaso Schirinzi, Enrico Silvio Bertini, Enrico Castelli, Gessica Vasco, Maurizio Petrarca

**Affiliations:** 1 Movement Analysis and Robotics Laboratory (MAR Lab), Intensive Neurorehabilitation and Robotics Department, “Bambino Gesù” Children’s Hospital, IRCCS, 00050 Rome, Italy; alberto.romano01@ateneopv.it (A.R.); martina.favetta@opbg.net (M.F.); enrico.castelli@opbg.net (E.C.); gessica.vasco@opbg.net (G.V.); maurizio.petrarca@opbg.net (M.P.); 2 Department of Systems Medicine, University of Rome “Tor Vergata”, 00133 Rome, Italy; t.schirinzi@yahoo.com; 3 Unit of Neuromuscolar and Neurodegenerative Diseases, Department of Neurosciences, “Bambino Gesù” Children’s Hospital, IRCCS, 00146 Rome, Italy; enricosilvio.bertini@opbg.net

**Keywords:** ataxia, exergaming, telerehabilitation, hand dexterity, treatment adherence and compliance

## Abstract

Background: Children with ataxia experience balance and movement coordination difficulties and needs intensive physical intervention to maintain functional abilities and counteract the disorder. Exergaming represents a valuable strategy to provide engaging physical intervention to children with ataxia, sustaining their motivation to perform the intervention. This paper aims to describe the effect of a home-conducted exergame-based exercise training for upper body movements control of children with ataxia on their ataxic symptoms, walking ability, and hand dexterity. Methods: Eighteen children with ataxia were randomly divided into intervention and control groups. Participants in the intervention group were asked to follow a 12-week motor activity program at home using the Niurion^®^ exergame. Blind assessments of participants’ ataxic symptoms, dominant and non-dominant hand dexterity, and walking ability were conducted. Results: On average, the participants performed the intervention for 61.5% of the expected time. At the end of the training, participants in the intervention group showed improved hand dexterity that worsened in the control group. Conclusion: The presented exergame enhanced the participants’ hand dexterity. However, there is a need for exergames capable of maintaining a high level of players’ motivation in playing. It is advisable to plan a mixed intervention to take care of the multiple aspects of the disorder.

## 1. Introduction

Ataxia refers to a group of motor disorders associated with the cerebellum or its afferent and efferent projections dysfunction or damage [1]. People with ataxia experience a lack of balance and movement coordination that leads to difficulties in walking and standing, poor limbs and fine hand function control, muscle tone alterations, dysarthria, and altered ocular motor function [2,3]. Children with ataxia show similar sensorimotor impairment as adults [4]. A recent literature review estimated a prevalence of 26/100,000 different forms of ataxia in European children [5]. The impairments derived from ataxia are especially debilitating during childhood as motor development, and learning processes are still ongoing [4]. Moreover, age is likely to affect engagement and compliance with the chosen intervention modality and may impact the targeting and timing of rehabilitation efforts. Children have different information-processing capacities compared to adults and respond differently to motor learning and skill-acquisition paradigms, suggesting children may require more exercise practice time before learning is consolidated [6].

As no effective curative treatments are available, exercise and physical therapy represent the core interventions available to these children [7,8]. Physical treatment should start as soon as the diagnosis of ataxia is given, even if only mild symptoms are present. Although the effectiveness of physical therapy intervention for children with ataxia is still not established [9,10], the therapeutic scenario might rapidly change because of the upcoming disease-modifying treatments or other symptomatic and rehabilitative interventions [9,10,11,12,13,14]. Moreover, a growing body of literature emerged in the last decade regarding exergames usage to provide physical therapy interventions to young patients with ataxia. The term exergame refers to digital games that require bodily movements to play, stimulating an active gaming experience to function as a form of physical activity [15,16,17]. Ilg and colleagues used three Microsoft Xbox Kinect (MXK) videogames to improve the balance and gait quality of six children and four adults with several types of progressive spinocerebellar ataxia [18]. Similarly, Schatton et al. reported the use of Nintendo Wii and MXK games to increase the body balance of six children and four adults with different types of ataxia [19]. Both studies reported a reduction in the participants’ ataxia symptoms, particularly related to gait and balance. Despite these preliminary positive results, the efficacy of exergame providing physical therapy to children with ataxia was, to date, only mildly tested. Moreover, the available studies focused on balance and gait, overlooking other key ataxia symptoms such as upper-limb function. A recent literature review [9] identified a sole case study proposing an elbow movements dexterity training for a 5-year old girl who had undergone surgical resection of a cerebellar tumor [20]. The patient was asked to track the movements of a pseudo-random target on a computer screen using elbow joint flexion and extension for two weeks, 10 min a day. The authors described an improvement in the participant’s elbow and hand movement dexterity.

Exergames hold the potential to support the motivation of children with ataxia to perform therapeutic activities in an intensive way and in a meaningful context that was found fundamental to achieve improvements in ataxia symptoms [8,21].

This paper aims to describe the effect of a home-conducted exergame-based exercise training for upper body movements control of children with ataxia on their hand dexterity, ataxic symptoms, and walking ability.

## 2. Materials and Methods

### 2.1. Ethical Issues

The study was conducted according to the ethical principles of the Helsinki Declaration and local regulations. All details relating to the study procedure were discussed with the candidates’ parents, and an informed consent document was signed for all participants. Enrolment was voluntary, with participants not receiving any incentives, financial or otherwise, for participation. The Ethical Committee of the Bambino Gesù Children’s Hospital approved the study.

### 2.2. Participants

Eighteen children and adolescents (mean age: 11.6 ± 3.5 years; age range: 5.1–17.2 years) were enrolled in this study. The inclusion criteria for this study were the presence of a confirmed diagnosis of ataxia and the absence of any signs of inflammatory, vascular malformation, or tumor central nervous system disease. During the recruitment phase, all participants underwent a specialist medical examination that assessed their cognitive and motor aspects to ensure that they could carry out the tests provided in the study protocol. Patients presenting with intellectual disabilities were excluded from the current investigation. All the candidates met the inclusion criteria. Participants’ age, sex, and ataxia characteristics are presented in Table 1. Two participants (11.1%) were diagnosed with non-genetic ataxia. All participants followed an individual physical therapy treatment for one 45-min session per week. These interventions concerned the development of activities aimed at improving the control of gross and fine motor movements, balance in sitting, standing, and walking, and dexterity in skills related to the activity of daily living (ADL).

### 2.3. Procedure

**Figure 1:**
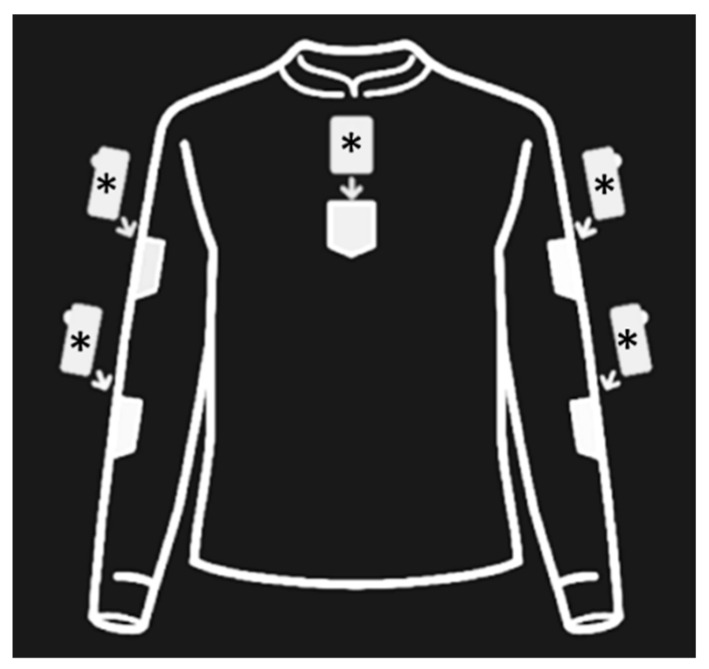
Visual description of IMU sensors (marked with asterisks) placement in the Niurion^®^ shirt.

All participants’ informed consents for participation were collected from their parents at the recruitment. Before starting the intervention (T0), all participants’ outcome measures were collected by two independent assessors with previous experience in the rehabilitation of people with ataxia. After the evaluation, the participants were randomly and consecutively assigned to the Intervention Group (IG) and Control Group (CG). The assessors did not know which group the participants were assigned at any study stage. Then, participants in the IG were given the exergame for upper body rehabilitation (Niurion^®^ kit—P2R, Bergamo, Italy). This inertial measurement unit (IMU) based rehabilitation device comprises five IMUs, a data receiver connected with a computer, an adherent shirt, and the software itself. The exergame included eight specific exercises aimed at improving the trunk and upper limbs movements control and muscle strength. Specific activities performed during each exercise were the following: elbows flexion, shoulders abduction at 90° and 180°, shoulders flexion at 90° and 180°, target reaching with the hand and arm in the ipsilateral and contralateral space, and anteroposterior trunk oscillation. Participants were asked to wear an adherent shirt and insert the IMUs in their designed pocket on the shirt (see Figure 1).

**Figure 2:**
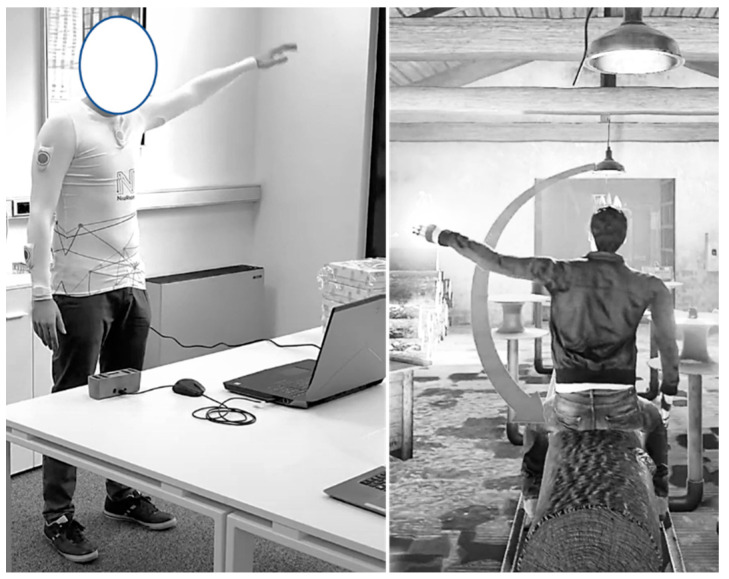
Example of shoulder 180° abduction exercise execution with related avatar movement.

Then, a calibration occurs, and an avatar reproducing the upper body movements of the participant was constructed by the software, and the exercises began. During each exercise, the participant stood in front of the screen and saw the avatar moving accordingly with his movements in the virtual space (see Figure 2).

Each exercise was performed in a different virtual space. The participant had to move his arms or body to interact with the environment and complete the game. The software was designed to recognize the trunk, the arm, and forearm movements allowing the interaction with the targets in the virtual environment. Moreover, the in-built algorithm of the software provides a real-time adaptation of the difficulty of the proposed tasks to avoid frustration due to continuous failures or motivation falling due to carrying out activities that are too simple for the subject. Each exercise lasted for seven minutes, and a 30 s recovery time was provided between the exercises (duration of the entire session: ~1 h). Each subject in the IG participated in two individual training sessions to be taught to use the system correctly. Participants’ parents also participate in these meetings to better comprehend the system’s functioning. At the end of the second training session, participants in the IG were asked to start the intervention at their home, performing the entire session (all the eight exercises) five days a week for 12 weeks (total of 60 sessions for each exercise). The time spent by each participant in the IG in the activities foreseen by the treatment was collected by the Niurion^®^ software (version 1.2.0, P2R, Italy) and analyzed to establish the participant’s adherence to the proposed intervention. Meanwhile, participants in the CG continued with the same therapeutic regimen conducted at a rehabilitation facility with their reference physiotherapist, without any change. None of the subjects in the IG changed their regimen of physical therapy sessions during the intervention period. Therefore, each participant received 12 physical therapy sessions within the duration of the current intervention.

At the end of the intervention (T1), the outcome measures were collected again for all participants, and obtained data were analyzed. Each participant’s number of therapeutic sessions was collected from their reference therapists at the end of the protocol.

### 2.4. Outcome Measure

The 9-Hole Peg Test (9HPT) was administered to obtain a timed measure of the participants’ pre- and post-intervention finger dexterity. This commonly used test requires placing and removing nine pegs in a pegboard as quickly as possible. The total time (seconds) to complete the task was recorded for both the dominant and non-dominant hands three times, and the mean of the three tests for each hand was calculated. This test has established intra- and inter-rater and test–retest reliability and normative reference values [22,23,24,25].

The Scale for the Assessment and Rating of Ataxia (SARA) was used to describe the ataxia severity level. Higher values reflect higher disease severity. This is a reliable and valid clinical scale measuring the severity of ataxia to be used in all cerebellar disorders [26,27,28]. SARA is “recommended” to assess cerebellar symptoms of different types of ataxia. It has been utilized by research groups other than the developer [3,29,30,31,32], and adequate psychometric proprieties support its use [33].

The Timed 25-Foot Walk test (T25FW) was used to assess the impact of the treatment on the participants’ global mobility ability. The test required the participant to walk a 25-foot-long path as fast as possible. The test was performed twice. The time (seconds) to complete the path was recorded for each trial, and the mean score was then calculated. The T25FW was found highly representative of mobility function and disability stage in patients with Friedreich ataxia [34,35].

### 2.5. Statistical Analysis

Due to the small sample size, the non-parametric statistic was used to analyze the collected data. The Mann–Whitney U test was first used to evaluate the comparability of participants’ age, sex, and the pre-intervention outcome measures scores among the IC and GC groups. The same test was conducted at the end of the intervention to compare the outcome measures variations between participants in the two groups. A delta was calculated with the difference between the scores obtained by each participant at T1 and T0 (∆ = T1 − T0) to obtain the outcome measures variations. The Wilcoxon Signed-Rank test was used to evaluate the variation that occurred in the outcome measures of participants of each group separately. The threshold for significance for the analyses above was assumed to be α = 0.05. No correction was applied for multiple comparisons [36].

## 3. Results

No difference was found between IG and CG when comparing the participants’ sex, age, and outcome measures scores at T0, proving the comparability of the two groups.

All the participants in the IG completed the protocol. The average adherence of each exercise is presented in Table 2. The average adherence was slightly varied among the exercises (range: 53.3–66.1%). All participants attended all the physical therapy sessions planned during the protocol (12 sessions). Individual participants’ adherence data and related descriptive statistics are available as Supplementary Material (Table S1).

The collected outcome measures scores and statistical analysis results are reported in Table 3. Participants’ individual scores for each outcome measure are available as Supplementary Material (Table S2). A significant change occurred in both groups’ dominant hand dexterity scores measured with the 9HPT at T1. Participants in the IG, on average, reduced the time needed to complete the task (representing an improvement—*p*: 0.05), while those in the CG increased it (representing a worsening—*p*: 0.03). In this test, the score changes that occurred in the IG and CG were statistically different (*p*: 0.01). The same trend was observable for the non-dominant hand. However, this difference did not reach the statistical significance between and within groups comparison analysis.

**Figure 3:**
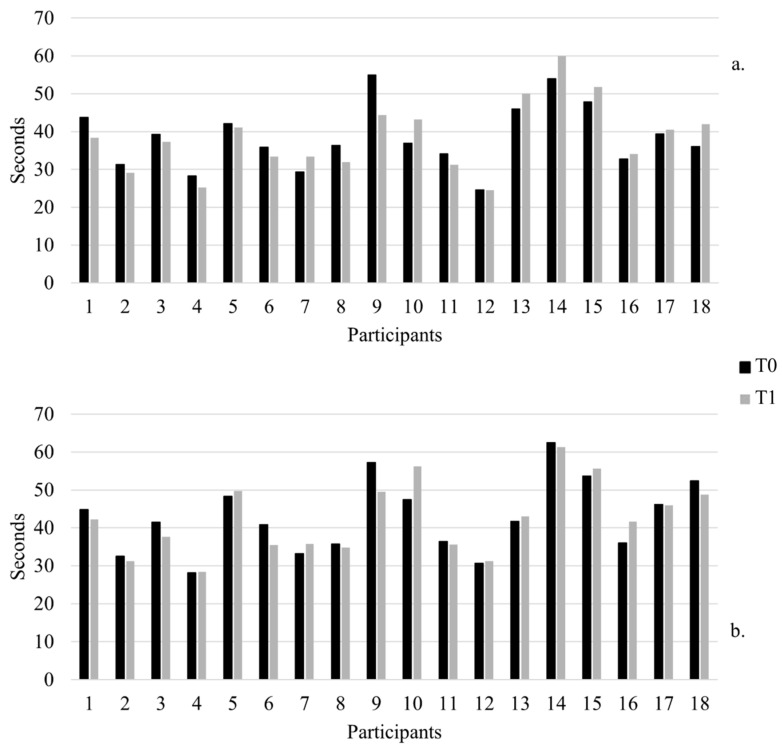
(**a**) Dominant and (**b**) non-dominant hands individual 9HPT scores for each participant at T0 and T1. Participants in the IG are numbered from one to nine; those in the CG are numbered from 10 to 18. Lower values represent better performance.

Participants’ individual data collected at T0 and T1 for each outcome measure are available as Supplementary Materials. Looking at the 9HPT scores, among the IG, improvements in the 9HPT scores were found in eight participants (88.9%) for the dominant hand and in six of them (77.8%) for the non-dominant hand. Conversely, in the CG, one subject (11.1%) ameliorated his dominant hand score, and four (44.4%) improved their performance with the non-dominant hand. Participants’ individual 9HPT scores are graphically represented in Figure 3.

No statistically significant changes were identified in the SARA items and total scores collected from the IG at T0 and T1. Conversely, a significant increment of the SARA total score was found for the CG at T1. However, although a more substantial change occurred in the CG, no statistical difference emerged comparing the SARA items and total score variation between the two groups.

No significant change was recognized when analyzing the T25FW scores. A minor improvement can be found in both groups at this test, with the CG reducing its scores slightly more than the IG.

## 4. Discussion

The present article described the effects of exergame use for upper body physical rehabilitation training for children with ataxia on participants’ hand dexterity, disease severity, and walking ability.

On average, the proposed intervention improved the hand dexterity of participants in the IG, while those in the CG worsened their performance. This result was confirmed when looking at the individual participants’ 9HPT scores showing that most of the subjects in the IG reduced their scores, and most of those in the CG increased the time required to complete the test. The 9HPT test–retest error margin was identified as 5% for the dominant hand and 2.4% for the non-dominant hand for the population with spinocerebellar ataxia [37]. Although minor changes occurred in the participants’ scores, the 9HPT deltas exceeded the test’s test–retest error margin in eight subjects for both the dominant and non-dominant hand in the IG. Of these, seven participants (77.8%) for the dominant hand and six (66.7%) for the non-dominant hand improved their score. Conversely, 9HPT deltas of six subjects for the dominant hand and five for the non-dominant hand surpass the test–retest error margin. Of them, five (55.6%) for the dominant hand and four (44.4%) for the non-dominant hand worsened their score. The authors believe that the conducted training strengthened the participants’ body and arms muscles and increased their body segments control, improving the trunk, shoulders, and arms stabilization during the hand dexterity task performance. These findings echo those of Ada and colleagues [20], who reported that computer-based elbow dexterity training positively affected the ipsilateral hand dexterity.

The disease severity data show that no significant change occurred for participants in the IC while those in the CG showed a significant increment of their SARA scores. This result should be interpreted with caution due to the small sample size. Moreover, although the disease progression resulted in diminished IC, no statistical difference was found between SARA score deltas of subjects in the IC and CG. Previous research reported better outcomes of exergame training on children’s ataxia symptoms assessed with the SARA [18,19]. However, these papers proposed treatments that focused more on balance and gait, while the one presented mainly involved arms movements. This discrepancy hints that a mixed intervention aimed at taking in charge different key aspects of the disorder may lead to more global improvements.

The walking ability changed accordingly in both groups showing no impact of the intervention on this skill. This result is not surprising as the implemented training was performed in a standing position and involved accurate upper limbs and trunk movements. Again, keeping in mind the globality of the motor impairment of children and adolescents with ataxia, it is advisable to combine interventions to take care of as many critical aspects of the pathology as possible. This finding is in line with a previous report suggesting that postural control can be enhanced using exergame as complementary training in adults with Ataxia [38]. The use of various exergames involving different body parts and skills across the week could also increase the child’s interest, supporting the adherence to the program.

Adherence data suggest that the presented results can be attained even with a less intensive intervention than expected. The authors believe that the Niurion^®^ exergame supported the participants’ motivation to follow and adhere to the intervention, but it was insufficient to sustain the required adherence. These data align with previous statements reporting exergames’ difficulties in maintaining the player’s interest over long periods [17,39]. The literature recommends intensive, daily physical intervention for this population that could lead to even better results if reached [8,21,38]. Highly motivating virtual environments are needed to stimulate young users to adhere to long-lasting intensive practices and achieve ecologic and meaningful treatment [40]. The individuality of the motivational factors necessary to sustain prolonged treatments requires the possibility of pursuing the same therapeutic goal throughout multiple virtual environments and tasks, adapting to personal and age adequate preferences. Although previous studies suggested some factors supporting the children’s motivation to play an exergame [41,42], the implementation of such elements remains technically challenging. However, exergaming is a relatively young and constantly developing technology, and its real potential in health promotion is far from being expressed [17].

Finally, considerations should be made on the fact that the present intervention was conducted at home, with minimal remote supervision of the health care professionals. These options represent a great advantage for monitoring and treating people with a rare pathology such as ataxia, which is widely distributed across the territory, sometimes with limited access to rehabilitation facilities. Moreover, as the proposed training acts as a supplementary intervention for the individuals in the IG, it could be speculated that the obtained improvement could be strictly related to the increased amount of time spent by participants in performing therapeutic activities, according to the existing literature [8,21,38]. Therefore, exergames could represent a cost-effective solution to increase the number of therapeutic sessions received by children with ataxia. The exergames cost-effectiveness is still limited by the hardware and software development price. However, more widely affordable solutions are emerging, such as the one used in this study and others [17,43,44,45,46,47].

This study presents some limitations. Although a robust experimental design was applied, a small number of participants was enrolled, limiting the generalization of the results. Enrolling large samples of patients with rare disorders in clinical rehabilitation research is difficult. Greater collaboration between scientists, clinicians, and the association of patients and families is needed to enhance the research quality in this field [11]. The external validity of our results is also challenged by the lack of a baseline and wash-out phases. Future studies should establish these phases to confirm the cause/effect relationship between the intervention and the occurred changes and their maintenance after the treatment interruption. In addition, there is a lack in the literature related to the expected change in the 9HPT score for the pediatric population with ataxia, challenging the possibility of comparing the presented results with the natural history of these patients. Finally, the effect of the intervention on the participants’ activity of daily living was not assessed within the current project and could represent an interesting study of the efficacy of exergame.

## 5. Conclusions

The presented exergame effectively enhanced the hand dexterity of children with ataxia. However, there is a need for more engaging and fun exergames capable of maintaining a high level of players’ interest and motivation in playing. Moreover, it is advisable to plan a mixed intervention (eventually with more than one exergame) to take care of the multiple aspects of the disorder.

## Figures and Tables

**Table 1:** Participants’ group, age at baseline (T0), diagnosis, and SARA items and total scores. The horizontal line separates the data obtained from participants in the IG and CG.

Group	Pt.	Age at T0 (Years)	Sex	Diagnosis	SARA Scores								
					Gait	Stance	Sitting	Speech Disturbance	Finger Chase	Nose-Finger Test	Fast Alternating Hand Movements	Heel-Shin Slide	SARA Total Score
**IG**	1	14.9	F	Non-genetic ataxia	2	1	0	2	1	1	3	1	11
	2	10.2	M	Joubert’s ataxia	3	1	0	1	1	0	1	1	8
	3	10	F	ARCA2	3	0	0	1	1	1	3	1	10
	4	8.6	M	ARCA2	1	0	0	0	0	1	1.5	0.5	4
	5	15.5	F	ARCA2	1	0	0	2	1	1	3	1	9
	6	9.5	F	Friedreich’s ataxia *	2	2	1	0	1	1	3	2	11
	7	8.5	M	Friedreich’s ataxia *	2	2	0	1	1	1	1	2	10
	8	16.9	F	Friedreich’s ataxia *	2	2	0	2	0.5	1	0	1.5	9
	9	10.5	F	ARSACS *	2	1	0	1	1	1	2.5	2	10.5
**CG**	10	17.2	F	Non-genetic ataxia	1	1	0	1	1	1	1	2	8
	11	15.5	F	Joubert’s ataxia	1	1	0	1	1	1	0	0	5
	12	11.2	M	Joubert’s ataxia	1	1	0	0	1	1	1	1	6
	13	10.4	M	ARCA2	2	2	0	2	1	1	3	1.5	12.5
	14	7.7	M	ARCA2	2	2	0	2	1	1.5	3	3.5	15
	15	9	F	Friedreich’s ataxia *	3	3	0	1	1	1.5	3	2.5	15
	16	12.6	F	Friedreich’s ataxia *	1	2	0	1	0.5	1	0.5	1.5	7.5
	17	15.4	M	Friedreich’s ataxia *	3	2	1	0	1	1	1	3	12
	18	5.1	F	Ataxia telangiectasia *	2	2	0	1	1	0	3	1	10

Abbreviation list: IG = Intervention Group; CG = Control Group; Pt. = Participants; M = Male; F = Female; ARCA2 = Autosomal Recessive Cerebellar Ataxia 2; ARSACS = Autosomal recessive spastic ataxia of Charlevoix-Saguenay; SARA = Scale for the Assessment and Rating of Ataxia. *: progressive ataxia.

**Table 2:** Average adherence for each exercise and the whole treatment. The percentage represents the average portion of time spent by participants in the IG in each exercise compared to the time required by the protocol (60 1 h sessions).

Exercises	Adherence Avg (SD)
Elbow flexion	53.3 ± 0.4%
Shoulder 90° abduction	64.1 ± 0.2%
Shoulder 180° abduction	58.0 ± 0.3%
Shoulder 90° flexion	64.4 ± 0.3%
Shoulder 180° flexion	66.1 ± 0.3%
Ipsilateral target reaching	62.8 ± 0.3%
Controlateral target reaching	56.4 ± 0.3%
Trunk oscillation	63.9 ± 0.3%
General adherence	61.5 ± 3.9%

Abbreviation list: Avg = Average; SD = Standard Deviation.

**Table 3:** Descriptive statistics and statistical analysis of outcome measures scores for IC and CG at T0 and T1 and occurred scores variation (∆). “IG” (T0 and T1), “CG” (T0 and T1), and “∆ Scores” (∆ IG and ∆ CG) columns report the average score and standard deviation (in parenthesis) for each outcome measure. “*p*-value IG T0 vs. T1” and “*p*-value CG T0 vs. T1” columns report the results obtained from the Wilcoxon Signed-Rank test analyzing the scores difference between T0 and T1 for each group. “*p*-value ∆ IG vs. ∆ CG” column reports the results attained from the Mann–Whitney U test comparing the variation in the scores (∆) of the IG and CG.

		IG		*p*-Value IG T0 vs. T1	CG		*p*-Value CG T0 vs. T1	∆ Scores		*p*-Value ∆ IG vs. ∆ CG
		T0	T1		T0	T1		∆ IG	∆ CG	
SARA Scores	Gait	2 (0.7)	2.1 (0.9)	0.65	1.8 (0.8)	2 (0.5)	0.48	−0.1 (0.8)	−0.2 (1)	0.60
	Stance	1 (0.9)	1.1 (0.8)	0.32	1.8 (0.7)	1.6 (0.7)	0.16	−0.1 (0.3)	0.2 (0.4)	0.09
	Sitting	0.1 (0.3)	0 (0)	0.32	0.1 (0.3)	0.3 (0.7)	0.16	0.1 (0.3)	−0.2 (0.4)	0.09
	Speech disturbance	1.1 (0.8)	1.2 (0.8)	1.00	1 (0.7)	1.4 (1)	0.18	0 (0.5)	−0.4 (1)	0.30
	Finger Chase	0.8 (0.4)	0.8 (0.4)	0.32	0.9 (0.2)	1.1 (0.8)	0.59	−0.1 (0.2)	−0.2 (0.8)	0.90
	Nose-finger test	0.9 (0.3)	1 (0)	1.00	1 (0.4)	1.2 (0.8)	0.71	0 (0)	−0.2 (0.8)	1.00
	Fast alternating hand movements	2 (1.1)	1.9 (0.9)	1.00	1.7 (1.3)	1.8 (1.2)	0.32	0.1 (0.8)	−0.1 (0.2)	0.90
	Heel-shin slide	1.3 (0.6)	1.4 (0.6)	0.16	1.8 (1.1)	1.7 (1)	1.00	−0.1 (0.2)	0.1 (0.8)	0.80
	SARA Total score	9.2 (2.2)	9.6 (2.4)	0.18	10.1 (3.7)	11.1 (3.9)	0.02 *↓	−0.4 (0.9)	−0.9 (1)	0.31
T25FW		5.3 (1)	5.3 (0.5)	0.86	6.3 (1.4)	5.9 (1.2)	0.31	0.1 (0.8)	0.3 (0.7)	0.31
9HPT Dominant hand		37.9 (8.4)	34.9 (6)	0.05 *↑	39.1 (8.9)	41.9 (11)	0.03 *↓	3 (3.9)	−2.6 (3.1)	0.01 *
9HPT Non-dominant hand		40.2 (9)	38.3 (7.5)	0.17	45.2 (10.1)	46.6 (9.9)	0.37	1.9 (3.3)	−1.6 (3.9)	0.08

Abbreviation list: SARA = Scale for Assessment and Rating of Ataxia; T25FW = Timed 25-Foot Walk; 9HPT = 9-Hole Peg Test; IG = Intervention Group; CG = Control Group; T0 = pre-intervention evaluation; T1 = post-intervention evaluation. ∆: Delta; *: *p*-value ≤ 0.05; ↑: statistically significant change reflects an improvement; ↓: statistically significant change reflects a worsening.

## Data Availability

Data are available as Supplementary Materials (see Tables S1 and S2).

## Supplementary Materials

The following supporting information can be downloaded at: https://www.mdpi.com/article/10.3390/jcm11041065/s1, Table S1: Adherence individual data for each participant and related descriptive statistics; Table S2: Participants’ individual scores for each outcome measure.
