# Supplementary material for: Upper Body Physical Rehabilitation for Children with Ataxia through IMU-Based Exergame
Source: J Clin Med. 2022 Feb 18;11(4):1065. doi: 10.3390/jcm11041065 (PMC8876617; doi:10.3390/jcm11041065)
Supplement: Supplementary file 1 [file jcm-11-01065-s001.zip › Table S1.pdf]

Journal of
Clinical Medicine

Upper Body Physical Rehabilitation for Children with Ataxia Through IMU-Based Exergame
Supplementary materials

MDPI

Table S1. Adherence individual data for each participant and related descriptive statistics.

		**Exercises**								**Descriptive statistics for participant**			
	**Pt.**	**Elbow flexion**	**Shoulder 90° abduction**	**Shoulder 180° abduction**	**Shoulder 90° flexion**	**Shoulder 180° flexion**	**Ipsilateral target reaching**	**Controlateral target reaching**	**Trunk oscillation**	**Avg.**	**SD**	**Max**	**Min**
Participants	1	60.0%	56.7%	44.0%	51.7%	40.0%	50.1%	50.0%	30.0%	47.8%	9.6%	60.0%	30.0%
	2	5.0%	80.0%	80.0%	78.3%	77.8%	81.8%	80.0%	75.0%	69.7%	26.2%	81.8%	5.0%
	3	76.7%	70.0%	48.0%	75.0%	86.7%	54.5%	65.0%	80.0%	69.5%	13.1%	86.7%	48.0%
	4	41.7%	51.7%	42.0%	46.7%	44.4%	58.2%	33.3%	50.0%	46.0%	7.5%	58.2%	33.3%
	5	100.0%	100.0%	100.0%	100.0%	100.0%	100.0%	100.0%	100.0%	100.0%	0.0%	100.0%	100.0%
	6	91.7%	65.0%	86.0%	81.7%	84.4%	94.5%	86.7%	77.5%	83.4%	9.2%	94.5%	65.0%
	7	20.0%	35.0%	22.0%	33.3%	\	27.3%	26.7%	10.0%	24.9%	8.5%	35.0%	10.0%
	8	93.3%	90.0%	88.0%	88.3%	91.1%	85.4%	96.7%	82.5%	89.4%	4.4%	96.7%	82.5%
	9	18.3%	28.3%	12.0%	25.0%	4.4%	23.6%	26.7%	2.5%	17.6%	10.2%	28.3%	2.5%
Descriptive statistics for exercise	Avg.	56.3%	64.1%	58.0%	64.4%	66.1%	63.9%	62.8%	56.4%				
	SD	36.3%	23.9%	31.4%	26.1%	33.0%	28.1%	29.7%	34.9%				
	Max	100.0%	100.0%	100.0%	100.0%	100.0%	100.0%	100.0%	100.0%				
	Min	5.0%	28.3%	12.0%	25.0%	4.4%	23.6%	26.7%	2.5%				

Abbreviation list: Pt. = Participants; Avg. = Average; SD = Standard Deviation; Max = Maximum; Min = Minimum.
\: exercise not performed due to technical issues.
