# Supplementary material for: Upper Body Physical Rehabilitation for Children with Ataxia through IMU-Based Exergame
Source: J Clin Med. 2022 Feb 18;11(4):1065. doi: 10.3390/jcm11041065 (PMC8876617; doi:10.3390/jcm11041065)
Supplement: Supplementary file 1 [file jcm-11-01065-s001.zip › Table S2.pdf]

Table S2. Participants' individual scores for each outcome measure.

		SARA Scores																	
Group	Pt.	Gait		Stance		Sitting		Speech disturbance		Finger Chase		Nose-finger test		Fast alternating hand movements		Heel-shin slide		SARA Total score	
		T0	T1	T0	T1	T0	T1	T0	T1	T0	T1	T0	T1	T0	T1	T0	T1	T0	T1
	1	2	2	1	1	0	0	2	2	1	1	1	1	3	3	1	1	11	11
	2	3	3	1	1	0	0	1	1	1	1	0	0	1	1	1	1.5	8	8.5
	3	3	2	0	1	0	0	1	1	1	1	1	1	3	3	1	1	10	10
	4	1	1	0	0	0	0	0	0	0	0	1	1	1.5	1.5	0.5	0.5	4	4
IG	5	1	1	0	0	0	0	2	2	1	1	1	1	3	3	1	1	9	9
	6	2	3	2	2	1	0	0	1	1	1	1	1	3	1	2	2	11	12
	7	2	3	2	2	0	0	1	1	1	1	1	1	1	1.5	2	2	10	11.5
	8	2	3	2	2	0	0	2	1	0.5	1	1	1	0	1	1.5	2	9	11
	9	2	1	1	1	0	0	1	1	1	1	1	1	2.5	2.5	2	2	10.5	9.5
	10	1	2	1	1	0	0	1	1	1	1	1	1	1	1.5	2	2	8	9.5
	11	1	2	1	1	0	0	1	1	1	0	1	1	0	0	0	0	5	5
	12	1	2	1	1	0	0	0	0	1	1	1	1	1	1	1	1	6	7
	13	2	2	2	2	0	0	2	2	1	1	1	1.5	3	3	1.5	2	12.5	13.5
CG	14	2	2	2	2	0	0	2	3	1	1	1.5	1	3	3	3.5	3	15	15
	15	3	3	3	3	0	1	1	1	1	1	1.5	1	3	3	2.5	3	15	16
	16	1	2	2	1	0	0	1	1	0.5	1	1	1	0.5	0.5	1.5	2	7.5	8.5
	17	3	1	2	1	1	2	0	3	1	3	1	3	1	1	3	1	12	15
	18	2	2	2	2	0	0	1	1	1	1	0	0	3	3	1	1	10	10

Table S2. Continue.

Group	Pt.	T25FW		9HPT Dominant hand		9HPT Non-dominant hand	
		T0	T1	T0	T1	T0	T1
IG	1	6.4	6.1	43.7	38.3	44.8	42.3
	2	7.7	5.8	31.3	29.1	32.5	31.3
	3	4.6	4.9	39.3	37.3	41.5	37.6
	4	4.8	5.3	28.3	25.2	28.1	28.4
	5	5.2	5.4	42.1	41.0	48.3	49.7
	6	4.9	4.7	35.8	33.4	40.8	35.5
	7	4.7	5.5	29.4	33.4	33.2	35.8
	8	5.2	4.9	36.4	32.0	35.7	34.8
	9	4.6	4.8	54.9	44.4	57.2	49.5
CG	10	5.4	5.5	36.9	43.2	47.4	56.3
	11	5.2	5.1	34.1	31.3	36.4	35.6
	12	5.8	4.3	24.6	24.6	30.7	31.3
	13	7.9	7.4	46.0	50.0	41.7	43.0
	14	5.9	5.3	54.0	60.0	62.5	61.3
	15	6.9	6.8	47.9	51.8	53.7	55.7
	16	5.1	5.3	32.7	34.1	36.0	41.7
	17	9.1	7.9	39.4	40.5	46.2	45.9
	18	5.1	6.0	36.1	42.0	52.4	48.7

Abbreviation list: IG = Intervention Group; CG = Control Group; Pt = Participants; T0 = pre-intervention evaluation; T1 = post-intervention evaluation; SARA = Scale for the Assessment and Rating of Ataxia; T25FW = Timed 25-Foot Walk; 9HPT = 9-Hole Peg Test.
